# Advances in the treatment of mycoses fungoides and Sézary syndrome: a narrative update in skin-directed therapies and immune-based treatments

**DOI:** 10.3389/fimmu.2023.1284045

**Published:** 2023-10-05

**Authors:** Robert Stuver, Shamir Geller

**Affiliations:** ^1^ Lymphoma Service, Department of Medicine, Memorial Sloan Kettering Cancer Center, New York, NY, United States; ^2^ Dermatology Service, Department of Medicine, Memorial Sloan Kettering Cancer Center, New York, NY, United States

**Keywords:** CTCL, brentuximab vedodin, mogamulizumab, mycoses fungoides, sezary syndrome

## Abstract

Mycoses fungoides (MF) and Sézary syndrome (SS) are cutaneous T-cell lymphomas that are often challenging to manage given the absence of reliably curative therapies, at times high symptom burden with significant detriment to quality of life, and need for ongoing treatment for disease and symptom control. Recent developments in skin-directed treatments include optimizing the use of existing topical therapies, the introduction of known dermatological agents and treatment modalities for the specific treatment of MF/SS (such as mechlorethamine gel, calcineurin inhibitor creams, and photodynamic therapy), and novel local and topical agents. For advanced disease, dedicated clinical trials have translated to exciting progress, leading to the approval of brentuximab vedotin (2017) and mogamulizumab (2018) for relapsed MF/SS. Additional studies of other active systemic agents, including various cellular therapies, represent further attempts to add to the therapeutic armamentarium in treating MF/SS. In this review, we highlight these recent advancements, ranging from optimization of skin-directed therapies to the introduction of novel systemic agents. We focus on therapies approved in the preceding five years or under investigation in advanced-phase clinical trials.

## Introduction and therapeutic framework

The cutaneous T-cell lymphomas (CTCLs) comprise a group of non-Hodgkin T-cell lymphomas primarily presenting with cutaneous involvement, though with capability to involve nodal regions, blood, and visceral organs (1). Mycosis fungoides (MF) is the most common type of CTCL, accounting for nearly 50% of all primary cutaneous lymphomas. Sézary syndrome (SS), often thought of as a leukemic form of MF though in fact a distinct disease entity (2–4), is rare and accounts for <5% of all primary cutaneous lymphomas. While the presentation, symptom burden, and management of patients with MF/SS is highly heterogenous, most patients with MF/SS have a chronic natural history requiring multiple treatments with variable responses and durability (5). It is paramount to realize that in the absence of aggressive treatment modalities, namely allogeneic hematopoietic stem cell transplant (alloSCT), conventional treatments for MF/SS are not curative. Still, patients with MF/SS, especially those with early-stage disease, can have prolonged survival measured in decades despite repeated relapses (6, 7). Therefore, an overarching tenant of therapy is to not only maximize disease control but also provide long-term palliation of symptoms and avoid cumulative treatment-related toxicity.

The management of MF/SS generally matches disease stage and symptom burden. Taking into account various patient-, disease-, and provider-specific variables, skin-directed therapies are usually used for patch/plaque disease with limited skin involvement (stage IA, see staging (8)). Frequent agents include topical corticosteroids (9), topical bexarotene (10), and topical mechlorethamine hydrochloride (11). More extensive patch/plaque disease is commonly approached with phototherapy (specifically, narrow-band ultraviolet B [NB-UVB] or Psoralen with ultraviolet A [PUVA]) (12), or radiation as either targeted treatment for localized skin disease and total skin electron beam (TSEB) therapy for diffuse or recalcitrant cases, often at reduced doses compared to conventional dosing (13, 14). Immunomodulatory agents, such as oral bexarotene (15, 16) low-dose methotrexate (17), and interferon (18), might be considered at these timepoints as well. More advanced disease, including tumor-stage MF or those with extracutaneous disease, generally require systemic agents, including single-agent chemotherapy (19–22), romidepsin (23, 24), vorinostat (25), and pralatrexate (26, 27). We emphasize that the management of MF/SS is highly individualized and is best served by a multidisciplinary oncology and dermatology approach. Our formal management practices are described elsewhere (28).

Building on the above framework, in this review, we discuss recent (occurring within the preceding five years) developments in the treatment of MF/SS, focusing on agents with published data leading to recent regulatory approval or those being evaluated in advanced-phase clinical trials. We draw upon our clinical experience with these agents and reviewed published manuscripts on these therapies. In addition, we reviewed published abstracts on MF/SS occurring in the last five meetings of the American Society of Hematology, the American Society of Clinical Oncology, and the European Hematology Association in order to identify additional agents for inclusion.

## Advances in skin-directed therapies

Recent developments in skin-directed treatments include greater understating of the optimal use of the existing topical therapies for MF/SS, introduction of known topical dermatological agents and treatment modalities in patients with MF/SS, and combinations of known skin-directed treatments with other therapies in attempts to increase efficacy. Some novel topical agents have been introduced in recent years and may represent important expansions to the arsenal of skin-directed-therapies for MF/SS in the near future.

### Chlormethine gel

Chlormethine (CL) or mechlorethamine gel 0.016% was approved the United States (US) Food and Drug Administration (FDA) in 2013 for the treatment of adult patients with stage IA–IB MF. CL induces double-stranded DNA breaks, thereby inducing apoptosis in skin-homing malignant T-cells and suppressing the expression of DNA repair genes in malignant T cells in the skin (29). The recently reported real-world PROVe study assessed real-world efficacy of CL in daily clinical practice in the US (2). Most patients in the PROVe study were using CL in combination with other therapies. The study included 298 patients and confirmed that CL is an important treatment for patients with MF, reducing the severity of cutaneous lesions and improving quality of life. At 12 months post-treatment initiation, 45% of patients had responded, with peak response occurring at 18 months (30). CL can be continued as maintenance therapy (31), with a recent single-center report demonstrating a 65% progression-free survival rate with CL gel maintenance, with a median time to progression of 29.5 months (32). An open-label study assessing the efficacy and tolerability of CL 0.04% in patients with early stage MF who had not achieved complete remission (CR) with one year of daily CL gel 0.02% showed that increased CL dose and longer treatment can result in further clinical benefit, especially for recalcitrant lesions, with no increase in toxicities or skin adverse effects (33).

### Calcineurin inhibitors

The calcineurin pathway is often activated in MF, and the safety and activity of 1% pimecrolimus cream, a topical calcineurin inhibitor, was recently evaluated in a multicenter, phase II trial of 39 patients with early-stage MF (PimTo-MF) (34). In total, 56% of patients had an overall response, most of which were partial responses. This agent is well tolerated, with no patients requiring a dose reduction or discontinuation due to drug-related toxicity in this trial. Adverse events occurred in 33% of the patients, most commonly a transitory grade 1 mild burning or pruritus. This agent is not approved for use in the US or Europe but can be used off-label in individualized cases.

The safety of topical tacrolimus, an additional calcineurin inhibitor, was recently reviewed in a single-center retrospective study of patients with MF at the University of Pennsylvania. In 13 patients with MF receiving topical tacrolimus for other (non-MF) conditions, there was no worsening or recurrence of MF over several years of follow-up in the majority of patients (35). These findings are in line with other recent large cohort studies showing no strong evidence of associations between topical calcineurin inhibitor use and the risk of developing lymphoma (or other cutaneous malignancies) (36, 37).

### Radiation therapy

Conventional total skin electron beam therapy (TSEBT) has known efficacy in treating MF/SS, though due to the chronic nature of MF/SS, many patients relapse and might face limited options for re-radiation retreatment due to skin toxicity. Therefore, low-dose TSEBT with 10-12 Gy has largely replaced traditional TSEBT (30-36 Gy) in the treatment of MF/SS (13, 38). Low-dose TSEBT provides rapid and reliable reduction of disease burden in MF/SS patients, and it can be safely administered in multiple instances with an acceptable toxicity profile (13). A combination strategy of low-dose TSEBT with oral bexarotene is being explored in a clinical trial at our center (NCT05296304). For localized disease, recent reports show that single-fraction radiation therapy with a dose of 8 Gy in one fraction can provide a high rate of complete and durable responses at affected sites (39).

### Photodynamic therapy

Photodynamic therapy (PDT) utilizing photosensitizing agents (such as 5-aminolevulinic acid [ALA]) irradiated with a specific wavelength of light in the presence of oxygen is being explored in CTCL (40, 41). Limiting the use of ALA-PDT in CTCL is the poor tissue penetration of the photosensitizers, though new developments in the use of PDT for CTCL, such as innovative photosensitizers beyond ALA, more effective delivery methods deeper into lymphoma lesions, and novel prepping methods, are ongoing (42). In particular, topical synthetic hypericin, known to inhibit malignant T-cell proliferation and induce apoptosis, has both a tumoricidal effect as a stand-alone drug and is activated by visible light (43). The randomized, placebo-controlled, double-blind, phase 3 FLASH trial, comprised of 169 patients with early-stage MF, evaluated the efficacy of topical 0.25% hypericin ointment PDT for early-stage disease (44). After six weeks of treatment, hypericin PDT was more effective than placebo (index lesion response rate after one cycle: 16% vs 4%, p=0.04). Responses increased to 40% after two cycles and 49% after three cycles, and were seen in both patch and plaque lesions. Adverse events were primarily mild application-site skin reactions with no evidence of systemic absorption. This agent is not approved by the US FDA but may represent an additional consideration in the near future.

### Excimer laser

The excimer laser, a form of UV light that emits 308-nm light, has demonstrated efficacy in the treatment of MF (12). Compared to other phototherapy modalities, advantages of the excimer laser include shorter therapy duration, ability to direct treatment at distinct skin lesions, and low UV dose exposure (45). In one report of 72 patients with MF who were treated with 308-nm excimer laser, 74% achieved a CR after a mean 18.7 (SD: 7.4, range: 6–44) treatment sessions. Only 2.8% had no clinical response. Lower response rates were reported in patients with folliculotropic MF (CR in 25% and PR in 75%). Commonly described adverse events are erythema, first-degree burn, and pruritus (45).

### Other topical therapies

As malignant lymphocytes of MF and SS overexpress CD47, this transmembrane protein represents a possible candidate for targeted anti-CD47 therapies. TTI-621 (SIRPαFc) is a fusion protein that blocks the CD47-SIRPα interaction between malignant cells and macrophages (46). Direct intralesional administration of TTI-621 to skin lesions has been trialed with the rational to enhance both local and systemic antitumor effects. Efficacy can be high with intralesional treatment, with reported overall response rates of 34% (46, 47). Intralesional TTI-621 is well tolerated and has activity in adjacent or distal non-injected lesions, suggesting a systemic effect (47). In another small clinical trial, a topical inhibitor of p-STAT3 (WP1220), has shown demonstrable safety and significant efficacy in three patients with progressive MF (48). Whether these agents will move forward for regulatory approval is unclear.

## Advances in systemic therapies

Progress in advanced-stage disease has centered around mechanistic or targeted strategies as opposed to traditional cytotoxic chemotherapy (**Table 1**). While chemotherapy is effective in MF/SS, it is not curative and generally cannot be given over long durations due to cumulative toxicity.

**Table 1:** Select Advances in Systemic Therapies in MF/SS.

Drug	Mechanism	Approval^1^	ORR^2^	Duration	Notable Adverse Reaction(s)
brentuximab vedotin^49^	anti-CD30 ADC	R/R CD30+ MF after 1 prior syst. therapy	65.6%	TTNT: 14.2 m	peripheral sensory and motor neuropathy
mogamulizumab^58^	anti-CCR4 ab	R/R MF/SS after 1 prior syst. therapy	MF: 21% SS: 37%	MF: 13.1 m SS: 17.3 m	infusion-related reaction, rash (see text for discussion)
E7777^75,76^	recombinant IL2-diptheria toxin protein	No	36.2%	not reported	infusion-related reaction, capillary leak syndrome, visual impairment
lacutamab^78^	anti-KIR3DL2 ab	No	36%	13.8 m	peripheral edema
pembrolizumab^87^	anti-PD1 therapy	No, NCCN compendium	38%	not reached (median f/u 58 w)	rash/flare, immune-related toxicity
dimethyl fumarate^91^	NF-κB inhibition, among others	No	30.4% (best overall skin response)	not reported	diarrhea, abdominal pain

1. This column refers to approval status based on the United Stated Food and Drug Administration (FDA).

2. Response rates in MF are highly nuanced and depend on the response criteria used, as well as disease stage, type of lesion (i.e., patch, plaque, or tumor), and compartment (i.e., skin, blood, lymph nodes, or organ). We encourage direct consultation with the referenced publication to review response rates in detail.

ab, antibody; ADC, antibody drug conjugate; CR, complete response; MF, mycoses fungoides; NCCN, National Comprehensive Cancer Network; ORR, objective response rate; syst., systemic; TTNT, time to next treatment; w, weeks.

### Brentuximab vedotin

Brentuximab vedotin (BV), an anti-CD30 antibody drug conjugate, was approved by the US FDA and European Union for CD30-positive MF after prior systemic therapy in November 2017 based on the international randomized phase 3 ALCANZA trial (49). In this trial of 131 patients with CD30-positive CTCL (this trial also enrolled patients with primary cutaneous anaplastic large cell lymphoma) who had previously been treated, patients were randomized to receive BV or physician’s choice of methotrexate or bexarotene. The primary endpoint was an objective global response lasting at least four months (ORR4). A greater proportion of patients receiving BV achieved an ORR4 (56% vs. 13%, p<0.0001). In addition, progression-free survival (PFS) was significantly prolonged with BV (17.2 vs. 3.5 months; HR 0.181, 95% CI 0.101-0.324) and patient-reported reduction in symptom burden as measured by Skindex-29 (50) was significantly greater with BV. The duration of skin response in responders to BV was long at 20.6 months, and extended followup showed significantly longer time-to-next-treatment in the BV arm (14.2 vs. 5.6 months; HR 0.7, 95% CI 0.17-0.42) (51). Sub-analyses of the ALCANZA trial and other studies have shown the efficacy of BV even in those with variable CD30 expression (52–55). Real-world results are generally consistent with those of the ALCANZA trial (56).

The development of peripheral neuropathy is a significant limitation to ongoing BV use, occurring in two-thirds of patients in the ALCANZA trial and often resulting in dose modifications (52%) or permanent discontinuation (14%). While over half of patients (59%) experience complete resolution of symptoms, ongoing neuropathy does occur. An ongoing trial of BV at 0.9 mg/kg and 1.2 mg/kg is testing the efficacy of reduced doses in attempts to minimize neuropathy and extend durability of treatment (NCT03587844) (57).

Finally, a recently reported exploratory regimen of BV in combination with romidepsin (NCT02616965) appears safe and effective, with a reported ORR among 15 patients in a phase I trial of 64%. We have not used this regimen and await additional data.

### Mogamulizumab

Mogamulizumab, a monoclonal antibody against C-C chemokine receptor 4 (CCR4), is a second agent approved by the US FDA and European Union for the treatment of MF/SS after at least one prior therapy. Approval is based on the international phase 3 MAVORIC trial, comparing mogamulizumab in patients with relapsed or refractory (R/R) MF/SS versus vorinostat (58). In this study of 372 patients, mogamulizumab met the primary endpoint of PFS, with a median of 7.7 versus 3.1 months in the vorinostat group (HR 0.53, 95% CI 0.41-0.69). Mogamulizumab also increased ORR in those with MF (21% vs. 7%) and SS (37% vs. 2%). There was a notable compartmental effect, with greater efficacy in the blood (ORR: 68%) and skin (ORR: 42%) than the lymph nodes (ORR: 17%). Lasting, deep responses (for example, ORR12) can be seen, especially in those with SS (59). Infusion-related reactions (37%) and skin eruptions (25%) are the most common adverse events. Mogamulizumab-associated rash (MAR) is challenging to clinically distinguish from disease; thorough dermatopathology review and T-cell clonality testing needed (60, 61). Patients who develop an on-treatment rash have significantly longer survival, potentially due to a robust immune response and long-term immune control via benign, activated T-cells and macrophages (62, 63). Rash management is non-standard but can be mitigated with topical and systemic steroids (usually followed by a taper), and most patients can resume treatment if discontinuation is needed (61, 64). Methotrexate has been used as a steroid-sparing agent in mogamulizumab-associated rash (61).

Resistance to mogamulizumab has recently been associated with loss of CCR4 expression and emergence of *CCR4* genomic alterations (65). Additional efforts building upon a mogamulizumab backbone are ongoing. Examples include magrolimab, a first-in-class anti-CD47 antibody with known efficacy in B-cell lymphoma (66), in combination with mogamulizumab in a phase I/II study (NCT04541017), as well as mogamulizumab in combination with IL-21 expanded NK cells, which are capable of high antibody-dependent cell-mediated cytotoxicity in combination with monoclonal antibodies (NCT0488064) (67).

An important consideration in the use of mogamulizumab is that its use prior to alloSCT (emanating from literature on its use in adult T-cell leukemia/lymphoma) has been shown to increase the risk of steroid-refractory graft-versus-host-disease, non-relapse mortality, and overall mortality, likely due to a deleterious effect on non-malignant T cells such as regulatory T cells (68–71). Some experts avoid mogamulizumab prior to alloHCT (72), whereas others recommend a minimum washout period of at least 50 days to mitigate against this complication (69).

### E7777

Denileukin diftitox (marketed as ONTAK) is a recombinant fusion protein combining the cytotoxic and membrane-translocating domains of diphtheria toxin with human IL-2, targeting cells with high-expression of IL-2 receptor (including malignant T cells) and resulting in cell death. Based on a randomized, phase III, placebo-controlled trial, DD was previously approved by the FDA in 2008 for use in R/R CTCL in patients whose tumor expressed the CD25 component of the IL-2 receptor (73). Due to production issues related to bacterial expression and purification changes, DD was voluntarily withdrawn in 2014 and has not been available for clinical use since that time. Manufacturing improvements have resulted in a purified compound (E7777, marketed as Lymphir), which was approved in Japan in 2021 for the treatment of R/R CTCL. This agent is considered a new drug by the US FDA and is seeking approval through a multicenter, open-label, single-arm registrational trial (NCT01871727) (74, 75). In the primary efficacy population (n=69), E7777 resulted in ORR of 36% (8.7% CR), with relative quick time to response (median: 1.4 months) and potential for durability (DOR greater than 12 months in 20%) (75). Infusion reactions (9%) and capillary leak syndrome (10%) were the most common serious adverse events, and nine patients (13%) experienced an event related to visual impairment (no grade 3-5), all adverse events that were previously observed with Ontak (74). No new safety signals were seen with E7777. If approved, E7777 will be a novel, non-cross resistant option in R/R MF/SS.

### Lacutamab

Lacutamab is a first-in-class humanized monoclonal antibody targeting the transmembrane protein killer cell immunoglobulin-like receptor 3DL2 (KIR3DL2), which is highly expressed especially in SS (76). In an international dose-escalation and cohort expansion phase I trial of 44 patients with R/R CTCL, lacutamab resulted in an ORR of 36%, with a median DOR of 13.8 months. Responses were overall higher in those with SS (ORR 43%) (77). In seven patients with prior mogamulizumab treatment, six either responded or had stable disease. The most common adverse events were peripheral edema (27%) and fatigue (20%). Infusion reactions are not common. The TELLOMAK trial, an ongoing, international phase II effort (NCT03902184), is evaluating lacutamab in patients with R/R MF/SS in multiple cohorts with varied inclusion criteria (78, 79). Interim evaluation of the cohort of patients with SS (R/R after at least two prior therapies, including mogamulizumab) showed global ORR of 21.6% in 37 patients, with highest responses in the blood (ORR: 37.8%, CR: 21.6%) (78). These results are encouraging in a population previously exposed to mogamulizumab. Lacutamab has been granted FDA Fast Track designation and EMA PRIME designation. The phase II trial is ongoing and we await further results.

### Anti-PD1 therapy

Checkpoint blockade has revolutionized the treatment of numerous solid and hematologic malignancies, though has proved challenging in T-cell lymphomas (80). The inhibitory receptor programed cell death protein 1 (PD-1) and its ligand, programmed death-ligand 1 (PD-L1), are widely expressed by malignant T-cell lymphomas and surrounding nonmalignant T cells (81, 82), though PD-1 may function as a haploinsufficient tumor suppressor, and therefore checkpoint inhibitors have the potential to accelerate existing T-cell lymphomas (83). These concerns were borne out in a phase II trial of nivolumab in adult T-cell leukemia/lymphoma (ATLL), in which the first three patients experienced rapid progression after a single infusion (84, 85).

In CTCL, the results have been mixed. In a phase II trial of pembrolizumab in 24 patients with R/R MF/SS, the ORR was 38% with seven partial responses and two complete responses (86). With a median time of response follow-up of 58 weeks, the median duration of response (DOR) was not reached. Among 15 patients with SS, eight experienced worsening of erythema and pruritis soon after starting treatment (after the first cycle), though most were able to remain on treatment with supportive measures (topical steroids) and eventually achieve a response. This worsening was considered a flare reaction and was associated with high expression of PD-1 on circulating Sézary cells. In practice, differentiating flare from hyperprogression is challenging. Separate reports have shown that PD-L1 structural variants, which can be seen in large cell transformation of MF, may predict sensitivity to checkpoint blockade and could prompt consideration for pembrolizumab use in clinical practice (87). Small studies with only preliminary results are evaluating combinations of pembrolizumab with other agents, including pralatrexate and decitabine (88). A second anti-PD-1 agent, durvalumab, has been combined with lenalidomide in phase I study, appearing to be safe with modest activity (89).

Pembrolizumab (nor any other checkpoint inhibitor) is not approved by the FDA for the treatment of MF/SS, but has compendium listing by the National Comprehensive Cancer Network and can be used off-label. We use pembrolizumab in clinical practice in select occasions.

### Dimethyl fumarate

Dimethyl fumarate (DMF) is a small-molecule compound approved for use in the treatment of relapsing forms of multiple sclerosis. DMF has varied downstream effects, one of which is NF-κB inhibition, known to be a constitutively active anti-apoptotic transcription factor in CTCL (90). As such, DMF as an agent to restore apoptosis sensitivity was recently demonstrated in a phase II trial of 25 patients with R/R MF/SS (91). DMF was given in escalating doses over the course of nine weeks and continued for a total of 24 weeks. The clinically efficacy was modest, with only five patients achieving the primary endpoint of a decrease in modified Severity-Weighted Assessment Tool (mSWAT) score of at least 50% after 24 weeks. The best overall response in the skin was 30.4%. There was no appreciable change in quality of life or pruritis. The fate of this agent may depend on its ability in combination with additional agents to result in greater response rates. Adverse events of grade 3 or higher are rare with DMF, and the primary side effects are those of gastrointestinal nature.

### Other systemic therapies

Cellular therapies remain in early stages in T-cell lymphomas. Interim results of an ongoing study (NCT0450246) of an allogeneic CD70-targeting chimeric antigen receptor T-cell (CAR T-cell) has been reported (92). Patients with R/R peripheral T-cell lymphoma (PTCL) and CTCL were treated at various dose levels following fludarabine plus cyclophosphamide lymphodepletion. In an interim analysis of 15 patients, ORR at dose-level (DL) three was 71% (in three patients with CTCL treated with at DL3, two responses were observed). No dose-limiting toxicities, ≥ grade 3 cytokine release syndrome, or ≥ grade 3 immune effector cell-associated neurotoxicity syndrome were observed. Dose expansion is ongoing. Other targets have been explored to a lesser extent. A registration-directed phase II trial of AFM13, a CD30/CD16A bispecific antibody, in CD30-positive PTCL or transformed MF, has completed enrollment (NCT04101331) (93). Other agents, including CD7- and CD30-directed CAR T-cells, are in various stages of development but not approved for use (94–96). While promising, the role of cellular therapy in T-cell lymphomas is a bit unclear and depends on further investigation.

Other therapies that have been primarily studied in PTCL include romidepsin plus lenalidomide (97), romidepsin plus duvelisib (98), and ruxolitinib (99). Small patient numbers among CTCL cohorts make formal efficacy evaluation of these regimens challenging, though we would consider use in multiply relapsed or refractory disease.

We acknowledge the limitations of this narrative review as opposed to a systematic review or meta-analysis. We aim for the text to provide a broad overview of updates in this therapeutic space.

## Conclusions

The treatment of MF/SS, especially advanced-stage disease, is challenging given the absence of reliably curative therapies, potential for high symptom burden with significant impact on quality of life, and frequent need for ongoing systemic therapy. Still, great progress has been made in the last five years, most notably with the approval of BV and mogamulizumab, demonstrated efficacy of pembrolizumab, and ongoing exploration of E7777 and lacutamab. Multiple additional agents, including those for early-stage disease, are under investigation. Continued translation of pre-clinical findings on pathogenesis into therapeutic strategies remains a key tenant to further advance the management of this disease.

## Author contributions

RS: Conceptualization, Writing – original draft, Writing – review & editing. SG: Conceptualization, Writing – original draft, Writing – review & editing.

## References

[1] Alaggio R Amador C Anagnostopoulos I. The 5th edition of the world health organization classification of haematolymphoid tumours: lymphoid neoplasms. Leukemia (2022) 36(7):1720–48. doi: 10.1038/s41375-022-01620-2 PMC9214472 35732829

[2] Booken N Gratchev A Utikal J. Sézary syndrome is a unique cutaneous T-cell lymphoma as identified by an expanded gene signature including diagnostic marker molecules CDO1 and DNM3. Leukemia (2008) 22(2):393–9. doi: 10.1038/sj.leu.2405044 18033314

[3] van Doorn R van Kester MS Dijkman R. Oncogenomic analysis of mycosis fungoides reveals major differences with Sézary syndrome. Blood (2009) 113(1):127–36. doi: 10.1182/blood-2008-04-153031 18832135

[4] Campbell JJ Clark RA Watanabe R Kupper TS. Sézary syndrome and mycosis fungoides arise from distinct T-cell subsets: a biologic rationale for their distinct clinical behaviors. Blood (2010) 116(5):767–71. doi: 10.1182/blood-2009-11-251926 PMC2918332 20484084

[5] Quaglino P Maule M Prince HM. Global patterns of care in advanced stage mycosis fungoides/Sezary syndrome: a multicenter retrospective follow-up study from the Cutaneous LymphomInternational Consortium. Ann Oncol (2017) 28(10):2517–25. doi: 10.1093/annonc/mdx352 28961843

[6] Talpur R Singh L Daulat S. Long-term outcomes of 1,263 patients with mycosis fungoides and Sézary syndrome from 1982 to 2009. Clin Cancer Res (2012) 18(18):5051–60. doi: 10.1158/1078-0432.CCR-12-0604 PMC3857608 22850569

[7] Agar NS Wedgeworth E Crichton S. Survival outcomes and prognostic factors in mycosis fungoides/Sézary syndrome: Validation of the revised International Society for Cutaneous Lymphomas/European Organisation for Research and Treatment of Cancer staging proposal. J Clin Oncol (2010) 28(31):4730–9. doi: 10.1200/JCO.2009.27.7665 20855822

[8] Olsen E Vonderheid E Pimpinelli N. Revisions to the staging and classification of mycosis fungoides and Sézary syndrome: a proposal of the International Society for Cutaneous Lymphomas (ISCL) and the cutaneous lymphoma task force of the European Organization of Research and Treatment of Cancer (EORTC). Blood (2007) 110(6):1713–22. doi: 10.1182/blood-2007-03-055749 17540844

[9] Zackheim HS. Treatment of patch-stage mycosis fungoides with topical corticosteroids. Dermatol Ther (2003) 16(4):283–7. doi: 10.1111/j.1396-0296.2003.01639.x 14686970

[10] Breneman D Duvic M Kuzel T. Phase 1 and 2 trial of bexarotene gel for skin-directed treatment of patients with cutaneous T-cell lymphoma. Arch Dermatol (2002) 138(3):325–32. doi: 10.1001/archderm.138.3.325 11902983

[11] Lessin SR Duvic M Guitart J. Topical chemotherapy in cutaneous T-cell lymphoma: positive results of a randomized, controlled, multicenter trial testing the efficacy and safety of a novel mechlorethamine, 0.02%, gel in mycosis fungoides. JAMA Dermatol (2013) 149(1):25–32. doi: 10.1001/2013.jamadermatol.541 23069814 PMC3662469

[12] Olsen EA Hodak E Anderson T. Guidelines for phototherapy of mycosis fungoides and Sézary syndrome: A consensus statement of the United States Cutaneous Lymphoma Consortium. J Am Acad Dermatol (2016) 74(1):27–58. doi: 10.1016/j.jaad.2015.09.033 26547257

[13] Hoppe RT Harrison C Tavallaee M. Low-dose total skin electron beam therapy as an effective modality to reduce disease burden in patients with mycosis fungoides: results of a pooled analysis from 3 phase-II clinical trials. J Am Acad Dermatol (2015) 72(2):286–92. doi: 10.1016/j.jaad.2014.10.014 25476993

[14] Kamstrup MR Gniadecki R Iversen L. Low-dose (10-Gy) total skin electron beam therapy for cutaneous t-cell lymphoma: An open clinical study and pooled data analysis. Int J Radiat Oncol Biol Phys (2015) 92(1):138–43. doi: 10.1016/j.ijrobp.2015.01.047 25863761

[15] Duvic M Hymes K Heald P. Bexarotene is effective and safe for treatment of refractory advanced-stage cutaneous T-cell lymphoma: multinational phase II-III trial results. J Clin Oncol (2001) 19(9):2456–71. doi: 10.1200/JCO.2001.19.9.2456 11331325

[16] Duvic M Martin AG Kim Y. Phase 2 and 3 clinical trial of oral bexarotene (Targretin capsules) for the treatment of refractory or persistent early-stage cutaneous T-cell lymphoma. Arch Dermatol (2001) 137(5):581–93. 11346336

[17] Zackheim HS Kashani-Sabet M McMillan A. Low-dose methotrexate to treat mycosis fungoides: a retrospective study in 69 patients. J Am Acad Dermatol (2003) 49(5):873–8. doi: 10.1016/S0190-9622(03)01591-3 14576667

[18] Kaplan EH Rosen ST Norris DB. Phase II study of recombinant human interferon gamma for treatment of cutaneous T-cell lymphoma. J Natl Cancer Inst (1990) 82(3):208–12. doi: 10.1093/jnci/82.3.208 2104937

[19] Marchi E Alinari L Tani M. Gemcitabine as frontline treatment for cutaneous T-cell lymphoma: phase II study of 32 patients. Cancer (2005) 104(11):2437–41. doi: 10.1002/cncr.21449 16216001

[20] Duvic M Talpur R Wen S. Phase II evaluation of gemcitabine monotherapy for cutaneous T-cell lymphoma. Clin Lymphoma Myeloma (2006) 7(1):51–8. doi: 10.3816/CLM.2006.n.039 16879770

[21] Quereux G Marques S Nguyen JM. Prospective multicenter study of pegylated liposomal doxorubicin treatment in patients with advanced or refractory mycosis fungoides or Sézary syndrome. Arch Dermatol (2008) 144(6):727–33. doi: 10.1001/archderm.144.6.727 18559761

[22] Dummer R Quaglino P Becker JC. Prospective international multicenter phase II trial of intravenous pegylated liposomal doxorubicin monochemotherapy in patients with stage IIB, IVA, or IVB advanced mycosis fungoides: final results from EORTC 21012. J Clin Oncol (2012) 30(33):4091–7. doi: 10.1200/JCO.2011.39.8065 23045580

[23] Piekarz RL Frye R Turner M. Phase II multi-institutional trial of the histone deacetylase inhibitor romidepsin as monotherapy for patients with cutaneous T-cell lymphoma. J Clin Oncol (2009) 27(32):5410–7. doi: 10.1200/JCO.2008.21.6150 PMC2773225 19826128

[24] Whittaker SJ Demierre MF Kim EJ. Final results from a multicenter, international, pivotal study of romidepsin in refractory cutaneous T-cell lymphoma. J Clin Oncol (2010) 28(29):4485–91. doi: 10.1200/JCO.2010.28.9066 20697094

[25] Olsen EA Kim YH Kuzel TM. Phase IIB multicenter trial of vorinostat in patients with persistent, progressive, or treatment refractory cutaneous t-cell lymphoma. J Clin Oncol (2007) 25(21):3109–15. doi: 10.1200/JCO.2006.10.2434 17577020

[26] Horwitz SM Kim YH Foss F. Identification of an active, well-tolerated dose of pralatrexate in patients with relapsed or refractory cutaneous T-cell lymphoma. Blood (2012) 119(18):4115–22. doi: 10.1182/blood-2011-11-390211 22394596

[27] Foss F Horwitz SM Coiffier B. Pralatrexate is an effective treatment for relapsed or refractory transformed mycosis fungoides: A subgroup efficacy analysis from the PROPEL study. Clin Lymphoma Myeloma Leuk (2012) 12(4):238–43. doi: 10.1016/j.clml.2012.01.010 22542448

[28] Khan N Noor SJ Horwitz S. How we treat mycosis fungoides and sézary syndrome. Clin Adv Hematol Oncol (2021) 19(9):581. PMC9364355 34495021

[29] Guenova E Ortiz-Romero PL Poligone B Querfeld C. Mechanism of action of chlormethine gel in mycosis fungoides. J Eur Acad Dermatol Venereol (2023) 37(9):1739–48 doi: 10.1111/jdv.19237 37262305

[30] Kim EJ Guitart J Querfeld C. The PROVe study: US real-world experience with chlormethine/mechlorethamine gel in combination with other therapies for patients with mycosis fungoides cutaneous T-cell lymphoma. Am J Clin Dermatol (2021) 22(3):407–14. doi: 10.1007/s40257-021-00591-x PMC8068681 33656660

[31] Querfeld C Nelson WW Gor D. Maintenance and concomitant therapy use with chlormethine gel among patients with stage IA/IB mycosis fungoides-type cutaneous T-cell lymphoma (MF-CTCL): A real-world evidence study. Dermatol Ther (Heidelb) (2022) 12(12):2781–95. doi: 10.1007/s13555-022-00831-w PMC9674809 36284059

[32] Correia E Krishnasamy S Suriano JG. Response to chlormethine/mechlorethamine gel maintenance treatment regimen in patients with mycosis fungoides: A single-center retrospective study. Clin Lymphoma Myeloma Leuk (2022) 22(8):581–8. doi: 10.1016/j.clml.2022.02.002 35393251

[33] Querfeld C Kim YH Guitart J Scarisbrick J Quaglino P. Use of chlormethine 0.04% gel for mycosis fungoides after treatment with topical chlormethine 0.02% gel: A phase 2 extension study. J Am Acad Dermatol (2022) 87(1):209–11. doi: 10.1016/j.jaad.2021.06.896 34333079

[34] Ortiz-Romero PL Maroñas Jiménez L Muniesa C. Activity and safety of topical pimecrolimus in patients with early stage mycosis fungoides (PimTo-MF): a single-arm, multicentre, phase 2 trial. Lancet Haematol (2022) 9(6):e425–33. doi: 10.1016/S2352-3026(22)00107-7 35654076

[35] Weiner DM Clark AK Bhansali RS. Usage and safety of topical tacrolimus in patients with mycosis fungoides. Clin Exp Dermatol (2022) 47(6):1200–1. doi: 10.1111/ced.15162 35245947

[36] Huang HH Shen D Chan TC. Association between the use of topical calcineurin inhibitors and the risk of cancer among patients with atopic dermatitis: A nationwide, population-based, retrospective cohort study. Am J Clin Dermatol (2023) 24(5):799–808. doi: 10.1007/s40257-023-00787-3 37280416

[37] Arana A Pottegård A Kuiper JG. Long-term risk of skin cancer and lymphoma in users of topical tacrolimus and pimecrolimus: final results from the extension of the cohort study protopic joint European longitudinal lymphoma and skin cancer evaluation (JOELLE). Clin Epidemiol (2021) 13(1141):1153. doi: 10.2147/CLEP.S331287 PMC8721027 35002327

[38] Grandi V Simontacchi G Grassi T Pileri A Pimpinelli N. Short-term efficacy and safety of total skin electron beam therapy in mycosis fungoides: Systematic review and meta-analysis. Dermatol Ther (2022) 35(11):e15840. doi: 10.1111/dth.15840 36124354 PMC9786352

[39] Wang P Gilbert M Lim HW. Single-fraction radiation therapy for localized cutaneous T-cell lymphoma. Pract Radiat Oncol (2023) 13(4):346–50. doi: 10.1016/j.prro.2023.03.015 37040819

[40] Brumfiel CM Severson KJ Patel MH. Photodynamic therapy in refractory mycosis fungoides: A prospective, open-label study. Blood (2020) 136(Supplement 1):32–2. doi: 10.1182/blood-2020-141119 33493572

[41] Liu WT Wang HT Yeh YH Wong TW. An update on recent advances of photodynamic therapy for primary cutaneous lymphomas. Pharmaceutics (2023) 15(5):1328. doi: 10.3390/pharmaceutics15051328 37242570 PMC10223676

[42] Caccavale S Tancredi V Vitiello P. Photodynamic therapy as an effective treatment for cutaneous lymphomas. Pharmaceutics (2022) 15(1):47. doi: 10.3390/pharmaceutics15010047 36678676 PMC9861941

[43] Fox FE Niu Z Tobia A Rook AH. Photoactivated hypericin is an anti-proliferative agent that induces a high rate of apoptotic death of normal, transformed, and Malignant T lymphocytes: implications for the treatment of cutaneous lymphoproliferative and inflammatory disorders. J Invest Dermatol (1998) 111(2):327–32. doi: 10.1046/j.1523-1747.1998.00278.x 9699738

[44] Kim EJ Mangold AR Desimone JA. Efficacy and safety of topical hypericin photodynamic therapy for early-stage cutaneous T-cell lymphoma (Mycosis fungoides): the FLASH phase 3 randomized clinical trial. JAMA Dermatol (2022) 158(9):1031–9. doi: 10.1001/jamadermatol.2022.2749 PMC9301595 35857290

[45] Sosh D Hyde J Dulmage B. The efficacy of 308-nm excimer laser in the treatment of mycosis fungoides. Int J Dermatol (2023) 62(2):e92–3. doi: 10.1111/ijd.16050 35049052

[46] Xiao A Akilov OE. Targeting the CD47-SIRPα Axis: present therapies and the future for cutaneous T-cell lymphoma. Cells (2022) 11(22):3591. doi: 10.3390/cells11223591 36429020 PMC9688096

[47] Querfeld C Thompson JA Taylor MH. Intralesional TTI-621, a novel biologic targeting the innate immune checkpoint CD47, in patients with relapsed or refractory mycosis fungoides or Sézary syndrome: a multicentre, phase 1 study. Lancet Haematol (2021) 8(11):e808–17. doi: 10.1016/S2352-3026(21)00271-4 34627593

[48] Sokołowska-Wojdyło M Blazewicz I Olszweska B. A phase 1b study evaluating the safety and efficacy of topical administration of WP1220, an inhibitor of STAT3 activation, in stage I, II or III mycosis fungoides (MF). Blood (2019) 134(Supplement_1):5272–2. doi: 10.1182/blood-2019-125118

[49] Prince HM Kim YH Horwitz SM. Brentuximab vedotin or physician’s choice in CD30-positive cutaneous T-cell lymphoma (ALCANZA): an international, open-label, randomised, phase 3, multicentre trial. Lancet (2017) 390(10094):555–66. doi: 10.1016/S0140-6736(17)31266-7 28600132

[50] Chren M-M Lasek RJ Flocke SA Zyzanski SJ. Improved discriminative and evaluative capability of a refined version of skindex, a quality-of-life instrument for patients with skin diseases. Arch Dermatol (1997) 133(11):1433–40. doi: 10.1001/archderm.1997.03890470111018 9371029

[51] Horwitz SM Scarisbrick JJ Dummer R. Randomized phase 3 ALCANZA study of brentuximab vedotin vs physician’s choice in cutaneous T-cell lymphoma: final data. Blood Adv (2021) 5(23):5098–106. doi: 10.1182/bloodadvances.2021004710 PMC9153035 34507350

[52] Kim YH Prince HM Whittaker S. Response to brentuximab vedotin versus physician’s choice by CD30 expression and large cell transformation status in patients with mycosis fungoides: An ALCANZA sub-analysis. Eur J Cancer (2021) 148:411–21. doi: 10.1016/j.ejca.2021.01.054 PMC9347228 33794441

[53] Kim YH Tavallaee M Sundram U. Phase II investigator-initiated study of brentuximab vedotin in mycosis fungoides and Sézary syndrome with variable CD30 expression level: A multi-institution collaborative project. J Clin Oncol (2015) 33(32):3750–8. doi: 10.1200/JCO.2014.60.3969 PMC5089160 26195720

[54] Papadavid E Kapniari E Pappa V. Multicentric EORTC retrospective study shows efficacy of brentuximab vedotin in patients who have mycosis fungoides and Sézary syndrome with variable CD30 positivity. Br J Dermatol (2021) 185(5):1035–44. doi: 10.1111/bjd.20588 34137025

[55] Jagadeesh D Horwitz S Bartlett NL. Response to brentuximab vedotin by CD30 expression in non-hodgkin lymphoma. Oncologist (2022) 27(10):864–73. doi: 10.1093/oncolo/oyac137 PMC9526494 35948003

[56] Barta SK Liu N DerSarkissian M. Real-world treatment patterns and clinical outcomes with brentuximab vedotin or other standard therapies in patients with previously treated cutaneous T-cell lymphoma (CTCL): A retrospective chart review study in the United States. Blood (2022) 140(Supplement 1):5167–9. doi: 10.1182/blood-2022-162375 37919137

[57] Khan N Noor S Geller S. A phase II trial of reduced dose brentuximab vedotin for cutaneous T-cell lymphomas. Hematol Oncol (2021) 39(S2). doi: 10.1002/hon.123_2880

[58] Kim Y Bagot M Pinter-Brown L. Mogamulizumab versus vorinostat in previously treated cutaneous T-cell lymphoma (MAVORIC): an international, open-label, randomised, controlled phase 3 trial. Lancet Oncol (2018) 19(9):1192–204. doi: 10.1016/S1470-2045(18)30379-6 30100375

[59] Kim YH Khodadoust MS de Masson A. Patient characteristics of long-term responders to mogamulizumab: results from the MAVORIC study. Blood (2020) 136(Supplement 1):35–5. doi: 10.1182/blood-2020-141379 34649658

[60] Wang JY Hirotsu KE Neal TM. Histopathologic characterization of mogamulizumab-associated rash. Am J Surg Pathol (2020) 44(12):1666–76. doi: 10.1097/PAS.0000000000001587 32976123

[61] Hirotsu KE Neal TM Khodadoust MS. Clinical characterization of mogamulizumab-associated rash during treatment of mycosis fungoides or sézary syndrome. JAMA Dermatol (2021) 157(6):700–7. doi: 10.1001/jamadermatol.2021.0877 PMC8060888 33881447

[62] de Masson A Darbord D Dobos G. Macrophage-derived CXCL9 and CXCL11, T-cell skin homing, and disease control in mogamulizumab-treated CTCL patients. Blood (2022) 139(12):1820–32. doi: 10.1182/blood.2021013341 34905599

[63] Hu B Atrash S Cohen L. Mogamulizumab-associated rash (MAR) correlates with longer progression free survival in cutaneous T cell lymphoma (CTCL). Blood (2022) 140(Supplement 1):1488–90. doi: 10.1182/blood-2022-166887

[64] Akilov O Geskin L Ito T. TCL-127: impact of concomitant steroids on mogamulizumab efficacy in MAVORIC. Clin Lymphoma Myeloma Leuk (2020) 20:S252–3. doi: 10.1016/S2152-2650(20)30849-1

[65] Beygi S Duran GE Fernandez-Pol S. Resistance to mogamulizumab is associated with loss of CCR4 in cutaneous T-cell lymphoma. Blood (2022) 139(26):3732–6. doi: 10.1182/blood.2021014468 PMC9247360 35436328

[66] Advani R Flinn I Popplewell L. CD47 blockade by hu5F9-G4 and rituximab in non-hodgkin’s lymphoma. N Engl J Med (2018) 379(18):1711–21. doi: 10.1056/NEJMoa1807315 PMC8058634 30380386

[67] William BM Reneau JC Campbell A. A pilot phase I trial of IL-21 expanded ideal-donor natural killer (NK) cells in combination with mogamulizumab in patients with cutaneous T-cell lymphomas (CTCL) or adult T-cell leukemia/lymphomas (ATLL). Blood (2021) 138(Supplement 1):1388–8. doi: 10.1182/blood-2021-150543

[68] Sugio T Kato K Aoki T. Mogamulizumab treatment prior to allogeneic hematopoietic stem cell transplantation induces severe acute graft-versus-host disease. Biol Blood Marrow Transplant (2016) 22(9):1608–14. doi: 10.1016/j.bbmt.2016.05.017 27220263

[69] Fuji S Inoue Y Utsunomiya A. Pretransplantation anti-CCR4 antibody mogamulizumab against adult T-cell leukemia/lymphoma is associated with significantly increased risks of severe and corticosteroid-refractory graft-versus-host disease, nonrelapse mortality, and overall mortality. J Clin Oncol (2016) 34(28):3426–33. doi: 10.1200/JCO.2016.67.8250 27507878

[70] Inoue Y Fuji S Tanosaki R Fukuda T. Pretransplant mogamulizumab against ATLL might increase the risk of acute GVHD and non-relapse mortality. Bone Marrow Transplant (2016) 51(5):725–7. doi: 10.1038/bmt.2015.315 26691420

[71] Haji S Kiyasu J Choi I. Administration of an anti-CC chemokine receptor 4 monoclonal antibody, mogamulizumab, before allogeneic bone marrow transplantation for adult T-cell leukemia/lymphoma. Bone Marrow Transplant (2016) 51(3):432–4. doi: 10.1038/bmt.2015.254 26524267

[72] Cook LB Phillips AA. How I treat adult T-cell leukemia/lymphoma. Blood (2021) 137(4):459–70. doi: 10.1182/blood.2019004045 33075812

[73] Prince HM Duvic M Martin A. Phase III placebo-controlled trial of denileukin diftitox for patients with cutaneous T-cell lymphoma. J Clin Oncol (2010) 28(11):1870–7. doi: 10.1200/JCO.2009.26.2386 20212249

[74] Prince HMM Geskin LJ Akilov OE. Safety and Tolerability of E7777 (improved purity Denileukin diftitox [ONTAK]) in Patients with Relapsed or Refractory Cutaneous T-Cell Lymphoma: Results from Pivotal Study 302. Blood (2022) 140(Supplement 1):6577–8. doi: 10.1182/blood-2022-167564

[75] Foss FM Kim YH Prince HMM. Efficacy and Safety of E7777 (improved purity Denileukin diftitox [ONTAK]) in Patients with Relapsed or Refractory Cutaneous T-Cell Lymphoma: Results from Pivotal Study 302. Blood (2022) 140(Supplement 1):1491–2. doi: 10.1182/blood-2022-166916

[76] Hurabielle C Thonnart N Ram-Wolff C. Usefulness of KIR3DL2 to diagnose, follow-up, and manage the treatment of patients with sézary syndrome. Clin Cancer Res (2017) 23(14):3619–27. doi: 10.1158/1078-0432.CCR-16-3185 28119365

[77] Bagot M Porcu P Marie-Cardine A. IPH4102, a first-in-class anti-KIR3DL2 monoclonal antibody, in patients with relapsed or refractory cutaneous T-cell lymphoma: an international, first-in-human, open-label, phase 1 trial. Lancet Oncol (2019) 20(8):1160–70. doi: 10.1016/S1470-2045(19)30320-1 31253572

[78] Bagot M Kim YH Ortiz-Romero PL. Lacutamab in patients with advanced sezary syndrome: results from an interim analysis of the tellomak phase 2 trial. Blood (2022) 140(Supplement 1):3760–1. doi: 10.1182/blood-2022-160239

[79] Kim WS Zinzani PL Marin-Niebla A. LACUTAMAB IN PATIENTS WITH ADVANCED MYCOSIS FUNGOIDES (MF): EFFICACY RESULTS ACCORDING TO UPDATED LYMPH NODE (LN) CLASSIFICATION IN THE TELLOMAK STUDY. Hematol Oncol (2023) 41(S2):196–7. doi: 10.1002/hon.3163_127

[80] Neuwelt A Al-Juhaishi T Davila E Haverkos B. Enhancing antitumor immunity through checkpoint blockade as a therapeutic strategy in T-cell lymphomas. Blood Adv (2020) 4(17):4256–66. doi: 10.1182/bloodadvances.2020001966 PMC7479955 32898250

[81] Wilcox RA Feldman AL Wada DA. B7-H1 (PD-L1, CD274) suppresses host immunity in T-cell lymphoproliferative disorders. Blood (2009) 114(10):2149–58. doi: 10.1182/blood-2009-04-216671 PMC2744574 19597183

[82] Krishnan C Warnke RA Arber DA Natkunam Y. PD-1 expression in T-cell lymphomas and reactive lymphoid entities: potential overlap in staining patterns between lymphoma and viral lymphadenitis. Am J Surg Pathol (2010) 34(2):178–89. doi: 10.1097/PAS.0b013e3181cc7e79 PMC2819320 20087161

[83] Wartewig T Kurgyis Z Keppler S. PD-1 is a haploinsufficient suppressor of T cell lymphomagenesis. Nature (2017) 552(7683):121–5. doi: 10.1038/nature24649 PMC5821214 29143824

[84] Ratner L Waldmann TA Janakiram M Brammer JE. Rapid progression of adult T-cell leukemia–lymphoma after PD-1 inhibitor therapy. New Engl J Med (2018) 378(20):1947–8. doi: 10.1056/NEJMc1803181 29768155

[85] Rauch DA Conlon KC Janakiram M. Rapid progression of adult T-cell leukemia/lymphoma as tumor-infiltrating Tregs after PD-1 blockade. Blood (2019) 134(17):1406–14. doi: 10.1182/blood.2019002038 PMC6839957 31467059

[86] Khodadoust MS Rook AH Porcu P. Pembrolizumab in relapsed and refractory mycosis fungoides and sézary syndrome: A multicenter phase II study. J Clin Oncol (2020) 38(1):20–8. doi: 10.1200/JCO.19.01056 PMC6943974 31532724

[87] Beygi S Fernandez-Pol S Duran G. Pembrolizumab in mycosis fungoides with PD-L1 structural variants. Blood Adv (2021) 5(3):771–4. doi: 10.1182/bloodadvances.2020002371 PMC7876893 33560388

[88] Roberts N Lister J Bennani NN. Pembrolizumab in combination with epigenetic therapy is safe and active in heavily treated patients with peripheral T-cell lymphoma (PTCL) and cutaneous T-cell lymphoma (CTCL): preliminary results from the embolden trial. Blood (2022) 140(Supplement 1):9425–6. doi: 10.1182/blood-2022-170181

[89] Querfeld C Tsai N-C Palmer J. Phase 1 results of anti-PD-ligand 1 (Durvalumab) & Lenalidomide in patients with cutaneous T cell lymphoma and correlation with programmed death ligand 1 expression and gene expression profile. Blood (2020) 136(Supplement 1):20–0. doi: 10.1182/blood-2020-143354

[90] Sors A Jean-Louis F Pellet C. Down-regulating constitutive activation of the NF-kappaB canonical pathway overcomes the resistance of cutaneous T-cell lymphoma to apoptosis. Blood (2006) 107(6):2354–63. doi: 10.1182/blood-2005-06-2536 16219794

[91] NicolayJPMelchersSAlbrechtJD. Dimethyl fumarate treatment in relapsed and refractory cutaneous T-cell lymphoma: a multicenter phase 2 study. Blood (2023) 142(9):794–805. doi: 10.1182/blood.2022018669 37217183

[92] Iyer SP Sica RA Ho PJ. S262: THE COBALT-LYM STUDY OF CTX130: A PHASE 1 DOSE ESCALATION STUDY OF CD70-TARGETED ALLOGENEIC CRISPR-CAS9–ENGINEERED CAR T CELLS IN PATIENTS WITH RELAPSED/REFRACTORY (R/R) T-CELL MALIGNANCIES. Hemasphere (2022) 6:163–4. doi: 10.1097/01.HS9.0000843940.96598.e2

[93] Choe-Juliak C Alexis KM Schwarz S Garcia L Sawas A. A phase II open-label multicenter study to assess the efficacy and safety of AFM13 in patients with relapsed or refractory CD30-positive peripheral T-cell lymphoma or transformed mycosis fungoides: The REDIRECT study design and rationale. J Clin Oncol (2020) 38(15S):163–4. doi: 10.1200/JCO.2020.38.15_suppl.TPS3148

[94] Zhang Y Li C Du M. Allogenic and autologous anti-CD7 CAR-T cell therapies in relapsed or refractory T-cell Malignancies. Blood Cancer J (2023) 13(1). doi: 10.1038/s41408-023-00822-w PMC10125858 37095094

[95] Grover NS Ivanova A Moore DT. CD30-directed CAR-T cells co-expressing CCR4 in relapsed/refractory hodgkin lymphoma and CD30+ Cutaneous T cell lymphoma. Blood (2021) 138(Supplement 1):742–2. doi: 10.1182/blood-2021-148102

[96] Gerlach M Schmitt S Cyprys P. Tub-010, a novel antibody-drug-conjugate with reduced nonspecific toxicity profile based on tub-tag technology widens the therapeutic window for the treatment of CD30+ Malignancies. Blood (2022) 140(Supplement 1):9397–8.

[97] Mehta-Shah N Lunning MA Moskowitz AJ. Romidepsin and lenalidomide-based regimens have efficacy in relapsed/refractory lymphoma: Combined analysis of two phase I studies with expansion cohorts. Am J Hematol (2021) 96(10):1211–22. doi: 10.1002/ajh.26288 PMC8811706 34251048

[98] Horwitz S Nikitina A Kotlov N. The combination of duvelisib and romidepsin (DR) is highly active against relapsed/refractory peripheral T-cell lymphoma with low rates of transaminitis: final results and biomarker analysis. Blood (2021) 138(Supplement 1):3847–7. doi: 10.1002/hon.56_2879

[99] Moskowitz AJ Ghione P Jacobsen E. A phase 2 biomarker-driven study of ruxolitinib demonstrates effectiveness of JAK/STAT targeting in T-cell lymphomas. Blood (2021) 138(26):2828–37. doi: 10.1182/blood.2021013379 PMC8718625 34653242

